# Exploring facilitating factors and barriers to the nationwide dissemination of a Dutch school-based obesity prevention program “DOiT”: a study protocol

**DOI:** 10.1186/1471-2458-13-1201

**Published:** 2013-12-19

**Authors:** Femke van Nassau, Amika S Singh, Willem van Mechelen, Theo GWM Paulussen, Johannes Brug, Mai JM Chinapaw

**Affiliations:** 1 Department of Public and Occupational Health, EMGO Institute for Health and Care Research, VU University Medical Center, Amsterdam, The Netherlands; 2 TNO (Netherlands Organisation of Applied Scientific Research) Life Style, Leiden, The Netherlands; 3 Department of Epidemiology and Biostatistics, EMGO Institute for Health and Care Research, VU University Medical Center, Amsterdam, The Netherlands

**Keywords:** Dissemination, Implementation, Adolescent, Overweight, Obesity, Schools, Prevention, Process evaluation

## Abstract

**Background:**

The evidence-based Dutch Obesity Intervention in Teenagers (DOiT) program is a school-based obesity prevention program for 12 to 14-year olds attending the first two years of prevocational education. This paper describes the study protocol applied to evaluate (a) the nationwide dissemination process of DOiT in the Netherlands, and (b) the relationship between quality of implementation and effectiveness during nationwide dissemination of the program in the Netherlands.

**Methods:**

In order to explore facilitating factors and barriers for dissemination of DOiT, we monitored the process of adoption, implementation and continuation of the DOiT program among 20 prevocational schools in the Netherlands. The study was an observational study using qualitative (i.e. semi-structured interviews) and quantitative methods (i.e. questionnaires and logbooks). Eight process indicators were assessed: recruitment, context, reach, dosage, fidelity, satisfaction, effectiveness and continuation. All teachers, students and parents involved in the implementation of the program were invited to participate in the study. As part of the process evaluation, a cluster-controlled trial with ten control schools was conducted to evaluate the effectiveness of the program on students’ anthropometry and energy balance-related behaviours and its association with quality of implementation.

**Discussion:**

The identified impeding and facilitating factors will contribute to an adjusted strategy promoting adoption, implementation and continuation of the DOiT program to ensure optimal use and, thereby, prevention of obesity in Dutch adolescents.

**Trial registration:**

Current Controlled Trials ISRCTN92755979.

## Background

How health-promotion programs perform, when widely disseminated under real-life conditions, has rarely evaluated [1]. Often, most effort is invested in assessing “efficacy”, i.e. the program’s effectiveness under well-controlled conditions [1,2]. The actual effectiveness of a health-promotion program depends on its efficacy, but also on its reach, adoption, implementation and maintenance [3-6]. However, when introduced under less-controlled conditions, insight into factors influencing the implementation of these efficacious interventions is crucial for translation into practice and systematic planning of dissemination strategies [7,8]. The aim of a dissemination strategy is to facilitate the process of behavioural change among implementers (e.g. professionals such as teachers, doctors, nurses), expected to be critical for the effective delivery of the program to the final target population (e.g. students, patients) [7]. Therefore, it is essential to tailor a dissemination plan to its users in order to stimulate applicability and transferability of health-promotion programs [7-9]. As yet, exemplary programs that have developed and evaluated effectiveness of a dissemination strategy in such a systematic way are scarce [3,10].

The Dutch Obesity Intervention in Teenagers (DOiT) program is an example of a health-promotion initiative that was ready for nationwide dissemination. DOiT is a school-based obesity prevention program for students attending the first two years of prevocational education (about 12–14 year olds). The DOiT program targets energy balance-related behaviours (EBRBs) in order to prevent overweight and obesity [11]. The program was developed using the Intervention Mapping (IM) protocol. Intervention Mapping is a protocol for developing health promotion interventions. Guided by six steps, the protocol supports intervention developers to identify and specify objectives, methods, and strategies regarding development, evaluation and implementation of interventions and programs [7].

DOiT showed promising effects on anthropometric measures (thinner skinfold thickness in girls and smaller waist circumference in boys) and EBRBs (a reduction in sugar-containing beverage consumption in both boys and girls and a reduction in screen-viewing time in boys) [12,13]. The accompanying process evaluation indicated that the majority of the students who were exposed to the program appreciated and used the DOiT materials and positively rated their experience with the program activities [14]. A majority of teachers regarded the DOiT materials as suitable for prevocational education. Teachers reported that they would recommend DOiT to other schools and planned to continue using DOiT themselves. Despite these positive findings, the evaluation exposed some impeding factors that hampered the implementation of DOiT. Most teachers needed more time than expected for the preparation and implementation of the lessons and they regarded the information contained in the materials to be too complex for the assumed student’s education level [14].

To prepare for wider dissemination in 2009, DOiT was adapted based on the teachers’ feedback from the former effect and process evaluation. A 7-step implementation strategy for DOiT was then developed in 2011 and a ‘DOiT support office’ was installed to guide and support the schools in their decision making, implementation and continuation with DOiT.

The objective of the described study was to evaluate the nationwide dissemination process of DOiT in order to gain insight into the facilitating factors and barriers to the nationwide dissemination of the DOiT program. Furthermore, the study aimed to evaluate the association between quality of implementation and effectiveness during the nationwide dissemination of the program in the Netherlands. We hypothesize that students attending schools with high quality of implementation will have better anthropometric and EBRBs outcomes, compared to students attending schools with lower quality of implementation. This article describes the study protocol applied for evaluation.

## Methods

**Figure 1:**
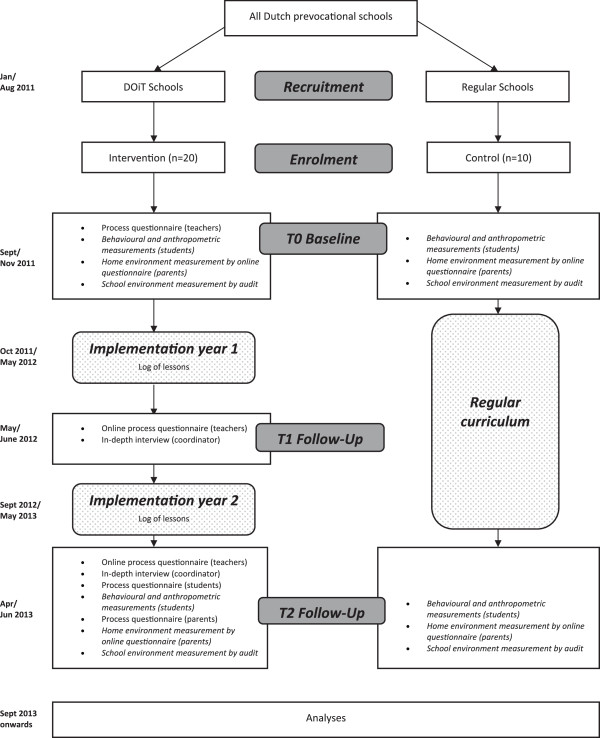
Flow chart of the DOiT study.

Data collection took place between January 2011 and June 2013. Data analyses are planned to take place from September 2013 onwards. Figure 1 provides a flow chart of the study.

### Study design and population

The study was an observational study using qualitative (i.e. semi-structured interviews) and quantitative methods (i.e. questionnaires and logbooks), allowing data triangulation to evaluate the dissemination process of DOiT at 20 prevocational education schools in the Netherlands. As part of the process evaluation of the dissemination, a cluster-controlled designed study with ten control schools, matched on level of education, location (rural/urban area) and school size, was conducted to evaluate the effectiveness of implementation of DOiT on anthropometrics and EBRBs of adolescent students. Students were recruited via the schools, so no individual contact information was needed. At each school, three classes were invited to participate, i.e. all students and their parents. At least two weeks prior to the commencement of the study, parents and students received an information letter explaining the background, aim and procedures of the study. There were no individual inclusion or exclusion criteria for study participation. When students moved to another school, they were not included in the follow-up measurement.

The Medical Ethics Committee of the VU University Medical Center approved the study protocol in which we applied for a passive consent procedure for parents, students and teachers. A passive consent procedure means that students or parents who did not want to participate in the study could send a letter to the researcher should they choose to decline participation. Students who declined for participation were excluded from the measurements. The study protocol adhered to the RATS guidelines.

### Dissemination plan and intervention

Since January 2011, the DOiT program has been available for schools in the Netherlands. This implies that all schools in the Netherlands can select to buy the DOiT program. In order to reach Dutch prevocational schools, a project employee is posted in the DOiT support office, located at the VU University Medical Center. The DOiT support office stimulates the process of adoption, implementation and continuation of DOiT throughout the school year as well as updating the DOiT website and responding to feedback. The DOiT support office actively recruits schools by activities such as posting news items on relevant websites, or in digital mailings and being present at national conferences and local meetings of relevant stakeholders. Additionally, the DOiT support office informs prevocational schools by sending a DOiT introductory package consisting of an information letter with a factsheet, a brochure and exemplary teaching materials. All schools in the Netherlands have free access to the implementation strategy and accompanying materials on the DOiT website. This evaluation is, therefore, an integral part of the on-going ’real life’ dissemination process of DOiT throughout the Netherlands.

#### DOiT program

The program was developed according to the Intervention Mapping protocol [7] using input from representatives of the target group (adolescents), implementers (teachers) and parents. DOiT is a school-based obesity prevention program for 12 to 14-year olds. DOiT focuses on five EBRBs: (1) reducing intake of sugar-containing beverages; (2) reducing intake of high-energy snacks; (3) reducing screen time; (4) increasing levels of physical activity (i.e. active transport and sports participation) and (5) daily and healthy breakfast consumption [11]. The program consists of a classroom component, an environmental component and a parental component. The program covers 12 fixed theory lessons, four physical education (PE) lessons, equally divided over two school years, and three optional additional lessons. The classroom lessons in the first year aim to increase awareness and knowledge of EBRBs. The lessons in the second year focus on the influence of the (obesogenic) environment. The environmental component aims to raise awareness of the school environment, finding solutions to reduce negative influences within the environment and setting a plan for improvement. The parental component focuses on stimulating social support of the parents and raising awareness of the availability and accessibility of healthy products and activities in the home environment. As part of the DOiT program, all parents receive an information booklet in which the topics of the DOiT lessons are described. During the program, students receive homework assignments to complete with their parents. Optionally, at the end of the program schools can organize a meeting for parents, where students present what they learned.

The DOiT materials include a ’schoolbook’ accompanied by worksheets, a student toolkit (pedometer, food/exercise diary and an online computer-tailored advice) and a parental information booklet. DOiT is supported by an extensive teacher manual with a login for extra materials provided at the DOiT website. Table 1 provides an overview of the DOiT program.

**Table 1:** Overview of the DOiT program

**Timeline**	**Classroom component**	**Environmental component**	**Parental component**
School year 1	**6 theory lessons:**		(1) Aimed at stimulating socialz support of the parents
	Aimed at raising awareness and information processing with regard to EBRBs		(2) Aimed at raising awareness of the availability and accessibility of healthy products and activities in the home environment
	**Materials:**		**Materials:**
	- 1 Textbook		- Information booklet
	- Online worksheets		- Homework assignments
	- Pedometer		- Information on DOiT website
	- Pocket-sized diary		- Optional parental meeting
	- Online computer-tailored advice		
	**2 PE lessons:**		
	Experiencing the acute effect of PA on the body measured by		
	(1) Pedometer		
	(2) Self-measured heart rate		
School year 2	**6 theory lessons:**	Aimed at raising awareness of the unhealthy environment, finding solutions and setting a plan for improvement of the environment	
	(1) Aimed at facilitation of choice to improve behaviour		
	(2) Aimed at raising awareness of the unhealthy environment, finding solutions and setting a plan for improvement of the environment	**Focus on:**	
		- Physical activity facilities in and around school	
		- Healthy school canteen	
		- (Un)healthy food retail outlets around school	
	**Materials:**		
			
			
	- 1 Textbook		
			
	- Online worksheets		
	- Small research		
	- Supportive video material		
	**2 PE lessons:**		
	(1) Experiencing the acute effect of PA on the body measured by self-measured heart rate		
	(2) Learn about sport possibilities in the neighbourhood		
Extra lessons	**3 optional additional lessons:**		
	(1) Cultural differences; learn about the cultural differences in food habits and physical activity		
	(2) Tasting; judging products by tasting, smelling and looking at (unfamiliar) snacks and soft drinks		
	(3) Cooking; preparing a healthy menu		

DOiT = Dutch Obesity Intervention in Teenagers; EBRB = Energy balance-related behaviour; PE = physical education; PA = physical activity.

#### Implementation strategy

Using the IM protocol, we developed the implementation strategy with the input of different implementation stakeholders. Teachers are the main implementers of DOiT. For that reason, teachers are supported by the DOiT implementation strategy. The strategy consists of seven steps to support adoption, implementation and continuation of DOiT within the school (see Table 2 for an overview). To facilitate the implementation process, a relatively low-profile implementation strategy with accompanying materials are available to teachers on the DOiT website [15]. The website provides general background information about the DOiT project, i.e. contact information, news items, information video, testimonials of schools who have successfully implemented DOiT, and a map of the Netherlands showing schools that currently implement DOiT. Teachers can read about DOiT and try the DOiT exemplary teaching materials in order to ensure that the program is compatible with their own context. The website has four domains targeting the different stakeholders i.e. 1) schools, 2) employees at supporting organisations, 3) parents and 4) students. In the parental domain, parents can read the information booklet online. Students have their own domain, containing a computer-tailored test and health-related videos. All domains are accessible free of charge.

**Table 2:** Implementation strategy and materials for implementation of DOiT by teachers

**Dissemination phase**	**Implementation strategy**	**Accompanying materials**
Adoption	Step 1. Teacher reviews the DOiT program	DOiT factsheet, brochure and exemplary teaching materials
	Step 2. Teacher identifies barriers for implementation, identifies solutions and gains support within the school	Example presentation for colleagues and school management
Implementation	Step 3. Teacher decides to work with DOiT and develops a tailored plan for implementation	Implementation plan: a checklist
	Step 4. Teacher becomes familiar with the implementation of the program	Example email to inform colleagues about the start of DOiT
		Example time line for implementation
		Instruction video
		Teacher manual
	Step 5. Teacher delivers the program	Example presentation for parents
		Template of press release
		Teacher manual
Continuation	Step 6. Teacher concludes and evaluates the program	Teacher manual
		Manual for parent meeting
	Step 7. Teacher defines impeding and facilitating factors for implementation and creates a renewed plan for implementation and embedding of the DOiT program in the school	Evaluation form
		Advice for continuation

Due to the ’real life’ condition of the study, there was no teacher training, interference or guidance by the research team.

### Framework for evaluation

In order to systematically evaluate the process of implementation at the schools, eight process indicators were assessed: recruitment, context, reach, dosage, fidelity, satisfaction, effectiveness and continuation. These process indicators were derived from the Diffusion of Innovation Theory of Rogers [6], the model developed by Steckler and Linnan [16], the Process Evaluation Plan of Saunders [17] and the RE-AIM framework [4,18]. Table 3 presents the process indicators and their definition stratified for the dissemination phases.

**Table 3:** Process evaluation indicators and their definition stratified for the three dissemination phases

**Process indicator**	**Definition**
**Adoption**	
Context	Factors of the physical, social, and political environment that either directly or indirectly affect the introduction of DOiT:
	a. Support within the schools (director and colleagues)
	b. School size; available budget; available hours for implementation of DOiT
	c. School environment (school canteen and sport facilities)
	d. Contamination with other programs aiming at a healthy lifestyle at school
	e. Teacher characteristics (e.g. knowledge, attitude, perception, willingness, self-efficacy, expectancy)
	f. Decision making process in the school
	g. Compatibility of the DOiT program with the regular curriculum
Recruitment	Exposure to sources and procedures applied for the recruitment of schools and teachers:
	a. Ways of approaching schools by the DOiT support office (used materials, message sent out)
	b. Ways of approaching schools by stakeholders (used materials, message sent out)
	c. Response of schools (reasons for agreement with participation, subgroups of recruited individuals or organisations, biases in response)
	d. Use of adoption materials
	e. Possible reasons for refusal or participation
Reach (1)	The extent to which the target population is reached by the recruitment strategy:
	a. Number of reached schools
	b. Number of reached stakeholders
**Implementation**	
Reach (2)	The extent to which the target population is reached by the implementation of DOiT:
	a. Number of teachers using DOiT
	b. Number of students using DOiT
	c. Number of parents reached by DOiT
Dosage	The proportion of DOiT lessons that were actually delivered or performed by the teachers and received by students:
	a. Implementation strategy activities that are accomplished
	b. Amount of DOiT lessons that are delivered or taught by teachers
	c. Completeness/delivery of implementation by the teachers (frequency, duration, mode of delivery, timing)
	d. Reasons for not delivering/implementing DOiT (facilitators/barriers for implementation)
Fidelity	The quality of the implementation of DOiT; the extent to which the teachers have implemented DOiT as intended by the developers:
	a. Compliance to the implementation strategy of DOiT (core elements)
	b. Compliance to the teacher manual of DOiT (core elements, standardisation)
Satisfaction	Subjective evaluation of DOiT and materials:
	a. General opinion about DOiT (by teachers, students and parents)
	b. Satisfaction with the DOiT program, materials, time spent and amount of lessons
	c. Satisfaction with implementation strategy, materials and support by DOiT support office
Effectiveness	The extent to which the DOiT program is effective:
	a. Behavioural and anthropometric change (students)
	b. Availability and accessibility of healthy products and activities in the home environment (parents)
	c. Availability and accessibility of foods and physical activity facilities in and around the school
**Continuation**	
Maintenance	The extent to which DOiT becomes routine and part of the curriculum and school policy:
	a. Embedding of DOiT in school health policy
	b. Embedding of DOiT in the curriculum of the school
	c. Future activities and intention to use DOiT
	d. Facilitators and barriers for future implementation

As part of the process evaluation, we assessed determinants that may explain the transition of the teachers through the subsequent stages of innovation: adoption, implementation and continuation. These determinants were categorized according to the innovation framework developed by Fleuren et al. [19]:

1) characteristics of the socio-political context, i.e. extent to which DOiT fitted into existing school health policy;

2) characteristics of the adopting organisation, i.e. decision making process in the school and school size;

3) characteristics of the teacher, i.e. teacher knowledge, skills, self-efficacy and intention to implement DOiT;

4) characteristics of the innovation, i.e. compatibility and flexibility of DOiT; and

5) characteristics of the dissemination strategy, i.e. training of the user and tools that influenced the implementation behaviour of the user.

The five determinant groups were operationalized into the process indicator context. The determinants will provide insight into factors that facilitate or impede the dissemination process of DOiT.

### Data collection procedures

#### Recruitment of schools

We aimed to include 20 DOiT implementation schools in the process evaluation. This number was based on feasibility and also a similar study, conducted in the Netherlands, evaluating the dissemination process of a Dutch healthy diet program, the Krachtvoer evaluation study [20].

After a school ordered the DOiT materials, the school was invited to participate in the study. The school was then informed about the study protocol by telephone and written information. If the school agreed to participate, the researcher visited the school in order to explain the procedures. Participating schools needed to meet the following criteria: (1) willing to implement DOiT during two subsequent school years; (2) a signed agreement of participation in the study during that period (2011–2013); (3) willingness to appoint a DOiT coordinator who was the linking agent between the research team and the school; (4) being able to participate with at least three classes of the first two years of their prevocational education, and (5) willingness to provide space and time for measurements. All teachers involved in the implementation of DOiT were asked to participate in the process evaluation. If schools participated in the study, they received free materials for three classes and a short report of the research results upon completion.

Regarding the process indicator effectiveness, 510 students per group were needed to detect a relevant difference in body mass index (BMI) between the intervention and control groups. Therefore, ten control schools were needed. Taking into account the clustered design, missing data and dropout (25%), a sample size of 1274 students were required to conduct multi-level analyses with an estimated power of 90% and an alpha level of significance set at 0.05 (see Table 4).

**Table 4:** Number of subjects needed to detect a relevant difference between DOiT and control schools

**Outcome**	**sd**	**Difference**	Number of subjects*	**Number of subject including 25% dropout**
BMI	1.2	0.25 kg/m^2^	510	637
Waist circumference	3.9	2 cm	80	100
Sum of skinfolds	14.6	5 mm	180	224
Sugar-containing beverages	840.4	250 ml/day	238	297
Sedentary behaviour	147.5	30 minutes/day	508	635
Active transport	23.3	10 minutes/day	115	143
Snacks	1.4	1 portion/day	40	51

*number of subjects required to conduct multi-level analyses with an estimated power of 90% and an alpha level of significance set at 0.05; sd = standard deviation based on the study of Singh et al. [12,13]; BMI = Body Mass Index.

In order to recruit control schools, we asked all intervention schools to provide the name of comparable schools in that area. As a result, we aimed to include control schools, matched on level of education, location (rural/urban area) and school size. Control schools did not implement DOiT during the study period. Regular contact with the management and teachers of control schools was ensured in order to promote their continued participation. Also the control schools received a short report of the research results upon completion.

#### Process measures

Process data were collected by teachers, DOiT coordinators, DOiT support office, students and parents throughout the whole research period.

#### Teachers

At baseline (T0), after eight (T1) and 20 months (T2), all teachers involved in the implementation of DOiT were asked to complete an online questionnaire about the impeding and facilitating factors of the implementation of DOiT. Completing the questionnaire took approximately 30 minutes. The majority of the questionnaire consisted of structured questions, measured on a bipolar five-point Likert scale. A few open-ended questions were added. The questionnaire was based on existing questionnaires, used in the previous DOiT evaluation [14,21] and the Krachtvoer evaluation study [22]. The questionnaire contained questions regarding the context, recruitment, satisfaction, maintenance, and potential relevant implementation-related determinants of the program. Additionally, the questionnaire contained items on other on-going studies and other innovations or school health-promotion programs. After each lesson teachers were requested to complete an online log. Completing the log took approximately 5 minutes per lesson. Using this log, we aimed to administer completeness of implementation by having teachers indicate if the lesson was taught, when it was taught and how the lesson was prepared and implemented. Teachers could tick off what activities were executed in preparation of the lesson on a list of proposed activities according to the teacher manual and how much time they spent on these activities. Teachers ticked off what materials were used during the lesson, what lesson activities were implemented and if the implementation complied with the prescription in the teacher manual. Finally, teachers reported how much time they spent in the classroom and rated their satisfaction with the lesson on a ten-point rating scale. Additionally to the questionnaires and logbooks, we aimed to visit each school at least once during the research period for a structured observation of a DOiT lesson.

#### DOiT coordinator at the schools

At the end of each school year (T1 and T2), i.e. in total twice during the research period, a sub-group of teachers and the coordinator were invited for an in-depth interview to gain more insight into the implementation of the DOiT program, its content, recruitment, fidelity and satisfaction with the dissemination strategy, and overall satisfaction with DOiT. Furthermore, facilitators and barriers for implementation, intentions and opportunities for future implementation of DOiT were discussed.

#### DOiT support office

During the recruitment period and the implementation period, the DOiT support office employee was requested to complete a log regarding all meetings, recruitment activities and communication with teachers and schools.

#### Non-adopting schools

After the recruitment phase, teachers that had not ordered the DOiT program were invited to complete a web-based questionnaire in order to gain insight into reasons for not implementing DOiT. The questionnaire contained questions regarding context and recruitment. Participants were invited by email to complete the questionnaire. Completing the questions took approximately 10 minutes.

#### Students

##### Questionnaire

Before the start of DOiT (T0) and after 20 months (T2), students were asked to complete the DOiT questionnaire in a classroom. The DOiT questionnaire addressed the following EBRBs: consumption of sugar-containing beverages; consumption of high sugar/high fat snacks; physical activity (sports and active transport); screen time (TV viewing and computer use) and breakfast behaviour. The questionnaire showed good test-retest reliability and moderate to good construct validity for the majority of items [23]. Since the item on physical activity (’hours of after school time physical activity at school’) showed a moderate test-retest reliability, we used the questions of the QAPACE questionnaire to assess physical activity [24]. Before the assessment, a researcher explained the procedures following a standardised protocol. Students needed approximately one school lesson (i.e. 45 minutes) to complete the questionnaire. After completing the measurements, students received a small incentive. At T2, we asked students to complete ten additional questions on their satisfaction with the program, their opinion about the content of the DOiT lessons and the layout of the DOiT materials. The questionnaire consisted of structured questions, measured on a ten-point rating scale.

##### Anthropometry

Before the start of DOiT (T0) and after 20 months (T2), we measured body weight, body height, skinfold thickness, waist and hip circumference. During PE lessons, anthropometric measurements were completed in the change-rooms. A separation structure was used to guarantee privacy during the measurements. The procedures of the measurements were explained to the students prior to any measurements being taken. Body weight was measured and recorded within 0.1 kg with a calibrated electronic flat scale (Seca 861), levelled after each placement. Body height was measured and recorded with an accuracy of 1 mm using a portable stadiometer. Skinfold thickness (i.e. triceps, biceps, suprailiac, subscapula) was measured on the left side of the body to the nearest 0.2 mm (Harpenden Skinfold Caliper) [25]. Two measurements were conducted. If the difference was more than 1 mm, a third measurement was taken. The skinfold thickness was taken as the average of the two nearest measurements. Both, waist and hip circumferences were measured with a Seca 206 waist circumference measure (Seca, Hamburg, Germany) to accuracy of 0.1 cm.

All student measurements (i.e. anthropometrics and questionnaire) were performed according to a standardised protocol by a trained research team during a 10-week period to minimise seasonal influences. The research team was not blinded to group assignment.

#### Parents

Before the start of DOiT (T0) and after 20 months (T2), parents were invited to complete an online questionnaire. Parents were reached via the school. Schools were asked to email the web link directly to parents. The questionnaire was based on the ENERGY parent questionnaire [26] and contained items on the physical home environmental, i.e. availability and accessibility of sugar-containing beverages and high sugar/high fat snacks, options for physical activity (sports and active transport), rules for screen time (TV viewing and computer use), availability and accessibility of breakfast products and availability of pocket money for food and drinks. The questionnaire mainly consisted of structured questions, measured on a bipolar five-point Likert scale. At T2, ten additional questions were asked about their satisfaction with DOiT (i.e. parental information booklet, homework assignments and parent meeting). Completing the questions took approximately 5 minutes.

#### School environment

Partly based on the audit instruments used in the ENERGY study [27] and the ENDORSE study [28], a DOiT audit instrument was developed to assess the availability and accessibility of foods and physical activity facilities in and around the school. Before the start of DOiT (T0) and after 20 months (T2), two researchers independently completed the audit instrument at each school. The audit instrument consisted of five parts: (1) food/drink available in the canteen; (2) food/drink available in vending machines; (3) physical activity facilities; (4) bicycle parking; and (5) available food retail outlets around school. For the majority of the items, the audit instrument had a ‘tick box’ answering format. The instrument assesses characteristics of the school environment in an objective manner. When subjective characteristics such as ‘state of baseball field’, or ‘space in the bicycle parking’ were present, photographs were taken. The DOiT audit instrument was pretested for its reliability at two schools.

### Analyses

Our first aim is to evaluate the nationwide dissemination process of DOiT in the Netherlands All qualitative data systematically collected or observed during the study will be considered as data [29,30]. This means that not only information from in-depth interviews with teachers, but also all data reported during phone calls or observed during school visits will be included in the analysis. Regarding the interviews, a literal transcript of the audiotape of each interview will be written. All transcripts and other data will be marked with codes (open coding). The codes will be grouped into similar concepts in order to make them more workable, i.e. selective coding. Using tables and matrices, we aim to identify and compare facilitators and barriers around the key process indicators and implementation-related determinants. All quantitative data obtained from the logbooks and the teacher questionnaires collected during the study will be analysed in SPSS 20.0 (SPSS Inc. Chicago, Illinois, USA).

Our second study aim is to examine the relationship between quality of implementation and changes in students’ EBRBs and anthropometrics. The change in students’ EBRBs and anthropometrics will be analysed using multi-level linear regression analysis. We define three levels in our multi-level analyses: 1) student, 2) class, and 3) school. Analyses will be adjusted for baseline values, age, gender, ethnicity, and educational level. These variables will be checked as potential effect modifiers. Multilevel statistical analyses will be performed using MLwiN 2.22.

The process data, including information on level of dosage, level of fidelity and quality of delivery, will be combined into an ‘school implementation score’. Using linear regression analysis, we will examine the association between quality of implementation (implementation score) and program effect on EBRBs, as well as on anthropometrics. For all analyses a two-tailed significance level of <0.05 will be considered statistically significant.

In addition, we will explore the behavioural mediators of the intervention effect on BMI, waist circumference and sum of skinfolds using the product-of-coefficient method of MacKinnon [31]. We will specifically look at the mediating effects of consumption of sugar-containing beverages; consumption of high sugar/high fat snacks; sports participation; active transport; screen time and breakfast behaviour.

## Discussion

This study investigates the barriers and facilitators of the dissemination process of DOiT in the Netherlands. The growing interest in translation of research into practice has created a need for evaluation of intervention implementation under real-life conditions [2].

Consequently, the main strength of this study is that we systematically monitored and evaluated the natural course of adoption, implementation and maintenance among implementers (i.e. teachers) at schools implementing DOiT [32]. The data of the process evaluation will provide insight into facilitators and barriers for successful dissemination of DOiT and therefore contribute to the translation of research into daily practice of health-promotion programs in the school-setting. Another strength of the study is that we monitored the effectiveness of the DOiT implementation in a longitudinal design, allowing analyses of effects on students’ EBRBs and anthropometrics, rather than cross-sectional associations only. If the data allows, both process and effect measures will help to explain the effectiveness of the program.

The study also has potential limitations. We chose to evaluate the implementation process under real-life conditions, thus the research team could not be blinded to group assignment and the schools could not be randomised. Since we evaluated the natural dissemination process, it is possible that adoption bias could have emerged. Rogers [6] states in the Diffusion of Innovation Theory, the rate of adoption is defined as the relative speed in which members of a social system adopt an innovation. It appears that early adopters are more willing to work with new innovations compared to the majority of the potential adopters. The decision to work with an innovation, such as DOiT, not only depends on its intrinsic value, such as demonstrated effectiveness, but also on perceived characteristics of the intervention, such as relative advantage. Since DOiT is an innovative program, teachers who adopt and implement DOiT may not be representative of all teachers of prevocational education in the Netherlands. This information needs to be taken into account when interpreting our results and optimising the implementation strategy of DOiT.

Although anthropometric measures (e.g. height, weight, skinfold thickness, hip and waist circumference) from all participating students were obtained objectively, students’ EBRBs and parental measures as well as teacher questionnaires and logbooks were self-reported and thus liable to social desirability and recall bias. We have selected the best available instruments with proven acceptable reliability and validity in our target group. If not available, we developed and pretested new or adjusted instruments.

We believe that the study with its focus on the dissemination process of DOiT, is a unique opportunity to gain insight into facilitating factors and barriers for implementation, overweight prevalence, associated EBRBs, and the home and school environment in the Netherlands. If the DOiT implementation strategy proves to be successful, we can use the study results to adapt this strategy. This strategy could also serve as an example for other programs, both national and international, providing prevocational schools with an effective implementation strategy for the implementation of health-promotion programs.

## Abbreviations

DOiT: Dutch obesity intervention in teenagers; EBRBs: Energy balance-related behaviours; PE: Physical education; sd: Standard deviation; BMI: Body mass index; PA: Physical activity.

## Competing interests

The authors declare that they have no competing interests.

## Authors’ contributions

All authors contributed to the design of the study. FvN was the principle researcher and was responsible for the data collection and coordinating the data collection. AS and MC were responsible for the conception of the study. TP advised on the design of the process evaluation of the study. AS, MC, WvM and JB supervised the study. All authors contributed to drafting this paper and approved the final manuscript.

## Pre-publication history

The pre-publication history for this paper can be accessed here:

http://www.biomedcentral.com/1471-2458/13/1201/prepub
